# Abnormal Reinnervation of Denervated Areas Following Nerve Injury Facilitates Neuropathic Pain

**DOI:** 10.3390/cells9041007

**Published:** 2020-04-18

**Authors:** Hodaya Leibovich, Nahum Buzaglo, Shlomo Tsuriel, Liat Peretz, Yaki Caspi, Ben Katz, Shaya Lev, David Lichtstein, Alexander M. Binshtok

**Affiliations:** 1 Department of Medical Neurobiology, Institute for Medical Research Israel Canada, Faculty of Medicine, The Hebrew University, Jerusalem 91120, Israel; 2 The Edmond and Lily Safra Center for Brain Sciences, The Hebrew University, Jerusalem 91120, Israel

**Keywords:** neuropathic pain, SNI, retrograde labeling, ouabain, reinnervation, denervation, sprouting, PNS, nociceptive, non-nociceptive

## Abstract

An injury to peripheral nerves leads to skin denervation, which often is followed by increased pain sensitivity of the denervated areas and the development of neuropathic pain. Changes in innervation patterns during the reinnervation process of the denervated skin could contribute to the development of neuropathic pain. Here, we examined the changes in the innervation pattern during reinnervation and correlated them with the symptoms of neuropathic pain. Using a multispectral labeling technique—PainBow, which we developed, we characterized dorsal root ganglion (DRG) neurons innervating distinct areas of the rats’ paw. We then used spared nerve injury, causing partial denervation of the paw, and examined the changes in innervation patterns of the denervated areas during the development of allodynia and hyperalgesia. We found that, differently from normal conditions, during the development of neuropathic pain, these areas were mainly innervated by large, non-nociceptive neurons. Moreover, we found that the development of neuropathic pain is correlated with an overall decrease in the number of DRG neurons innervating these areas. Importantly, treatment with ouabain facilitated reinnervation and alleviated neuropathic pain. Our results suggest that local changes in peripheral innervation following denervation contribute to neuropathic pain development. The reversal of these changes decreases neuropathic pain.

## 1. Introduction

Chronic neuropathic pain is characterized by hyperalgesia (hypersensitivity to noxious stimuli), allodynia (the sensation of pain to innocuous stimuli), and prolonged episodes of spontaneous pain [1,2]. Neuropathic pain is caused by damage to peripheral nerves or disease affecting them [2,3]. The abnormal activity of injured peripheral nerves triggers hyperexcitable changes in the central nervous system, known as central sensitization, which is the key factor in the development of neuropathic pain [4,5]. The injury of peripheral nerves also causes the denervation of body areas innervated by these nerves. However, these areas are subsequently reinnervated by peripheral nerves [6,7,8,9,10,11]. Importantly, reinnervation leads to changes in skin innervation patterns. For example, a behavioral study that examined changes in the mechanical threshold in denervated paw following a complete section of the sciatic nerve showed that reinnervation begins within a week after injury [12]. This study also suggests that reinnervation originated from a collateral sprouting of high-threshold mechanoreceptors from the intact saphenous nerve, which invaded the denervating skin, thus leading to the development of mechanical allodynia [12]. Histological and immunohistochemical studies on excised paw skins of rats following a loose ligation of the common sciatic nerve or spared nerve injury (SNI) demonstrate different patterns of reinnervation of denervated areas, depending on the type of nerve injury [7,8,9,13]. Importantly, it has been suggested that the changes in innervation patterns contribute to neuropathic pain [7,8,9,13].

Here, we aimed to study the innervation patterns following nerve injury, focusing on the changes at the level of neuronal somata at the dorsal root ganglions (DRGs). We examined whether these changes underlie the development of neuropathic pain. We have developed a multispectral retrograde labeling approach allowing for us to characterize the amount and type (nociceptive, non-nociceptive) of DRG neurons innervating specific areas (innervated by tibial and peroneal nerves) in the rat’s plantar hind paw skin to study the reinnervation of denervated areas at the level of the DRGs. In consideration with the multispectral nature of this approach, we called it Painbow. We then used the SNI model for neuropathic pain, which produces partial denervation of the paw, specifically at areas that are innervated by the tibial and peroneal nerves [14]. Thus, the SNI model allows for distinguishing between non-injured, innervated, and adjacent, denervated skin territories while preventing the co-mingling of distal intact axons with degenerating axons [14,15,16]. In the non-injured skin territories of the sural and the saphenous nerves, SNI results in early-onset (less than 24 h) and long-lasting allodynia and mechanical hyperalgesia. Importantly, the hypersensitivity to noxious and innocuous stimuli is also evoked from skin territories of the injured tibial and peroneal nerves after they have recovered from SNI-induced denervation [7,8,12,13]. In the current study, we followed the changes in the number and type of DRG neurons at different stages of SNI, i.e., during the denervation, the reinnervation, and the development of neuropathic pain. We found that, in normal conditions, the tibial and peroneal areas of the plantar hind paw skin are equally innervated by nociceptive and non-nociceptive cells. This ratio is disrupted during the development of neuropathic symptoms, such that these areas are primarily innervated by large non-nociceptive neurons. Moreover, we found that during the development of neuropathic pain symptoms, these areas are reinnervated by an overall significantly lower amount of DRG neurons. Treatment with ouabain, which facilitates sprouting by increasing the number of DRG neurons innervating tibial and peroneal skin areas, substantially reduced pain behavior. Our results suggest that the changes in innervation patterns during reinnervation contributes to the development of neuropathic pain. Moreover, our results suggest that ouabain or other treatments that promote reinnervation could be potentially beneficial in the treatment of neuropathic pain.

## 2. Materials and Methods

### 2.1. Animals

All of the procedures performed herein were in full accordance with the guidelines of the Hebrew University Animal Care Committee and were approved by the Committee. Procedures were performed on adult (200–250 g) male Naïve Sprague-Dawley rats. The rats were housed in groups of three rats under a 12-h light/dark cycle. Rats were habituated to the testing environment for at least three sessions. The experimenter was blind to the treatments. The room temperature and humidity remained stable during the entire experiment, and food and water were regularly provided.

### 2.2. Spared Nerve Injury (SNI)

The surgery was performed under ketamine anesthesia. Ketamine with xylazine (for muscle relaxation) were injected intraperitoneal (i.p.), with a 1:5 ratio of xylazine to ketamine. The initial injection consisted of 0.4 mL of the sedation mixture, with the addition of 0.1 mL as needed to ensure sedation throughout the whole procedure. The skin on the lateral surface of the thigh was incised, and a section through the biceps femoris muscle was made, exposing the sciatic nerve and its three terminal branches: the sural, the common peroneal, and tibial nerves. The SNI procedure included a ligation, followed by axotomy of the tibial and common peroneal nerves, leaving the sural nerve intact. The ligation was made with a 0–5 black braided string, and the incision was made distal to the ligation, removing 2–4 mm of the distal nerve stump. While performing the surgery, an effort was made to avoid any contact with or stretch of the intact sural nerve. Muscle and skin were stitched at the end of the ligation.

### 2.3. DRG Neuronal Labeling—Painbow

**Figure 1:**
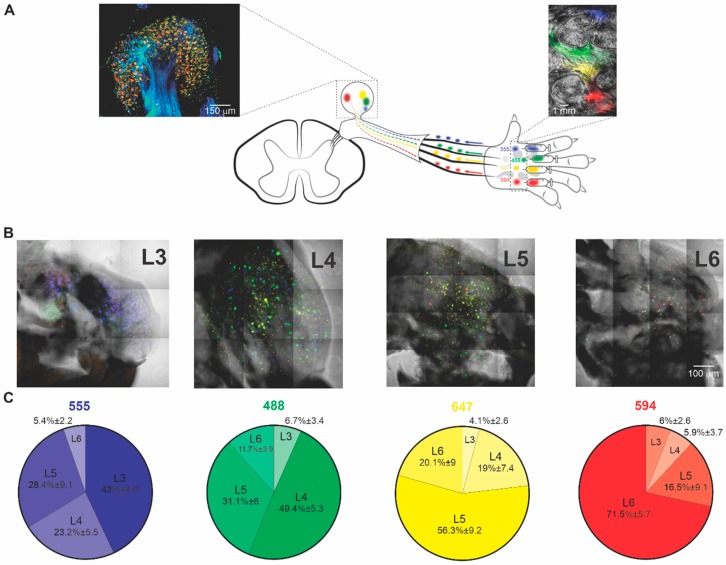
Retrograde labeling of dorsal root ganglion (DRG) somata that innervate distinctive areas of the paw. (**A**) Scheme depicting the Painbow approach. Four spectrally different fluorophores AF 488—green, AF 555—blue, AF 594—red, AF 647—yellow, conjugated to wheat germ agglutinin (WGA), were injected into the paw in the illustrated order. The dyes were uptaken by the axonal terminals innervating the injected paw skin and transported to DRG somata. The labeling of the specific DRG reflects the area in the paw this DRG innervates. Left inset, a high-resolution confocal fluorescent image of L_5_ DRG excised 40 h after injecting the dyes into the skin. Right inset, the fluorescent image of the dissected paw skin obtained 40 h after the dye injection. Note that colors remained in the paw 40 h after the injection, allowing the correlation between the skin area and labeled DRG somata. Note differently labeled somata of DRG neurons. (**B**) Collaged fluorescent photomicrography of representative individual L_3_-L_6_ DRGs excised from naïve rats. Colored dots are DRG somata, retrogradely labeled from their innervation targets. (**C**) Distribution of injected dyes among lumbar DRGs of naïve rats. Note that as expected form the innervation patterns, AF555 injected into the medial part of the paw is mainly present in L_3_ DRG; AF488 injected laterally to AF555 is mainly present in L_4_ DRG; AF647 injected into the middle of the paw is mainly present in L_5_ DRG, and AF594 injected into the lateral paw is mainly present in L_6_ DRG. Data from five DRGs for each level from 5 rats, chi-square independence test (9) = 236.58, *p* < 0.001, suggesting that the DRG segments and the distribution of the colors are not independent of each other.

**Figure 2:**
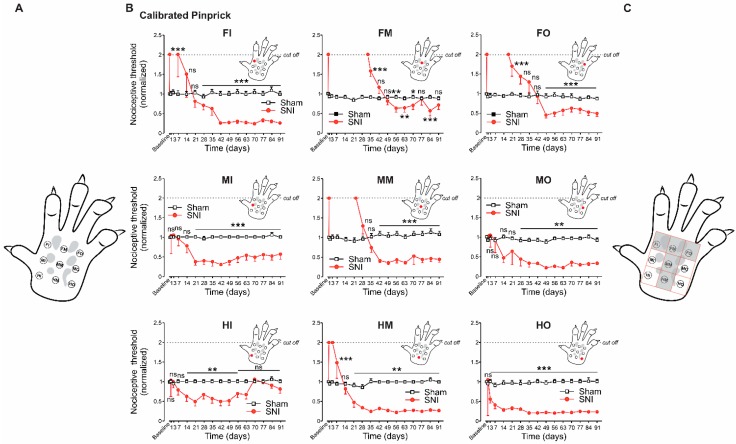
Mapping of the paw reveals differences in the time kinetics of denervation, reinnervation, and development of mechanical hyperalgesia following spared nerve injury (SNI). (**A**) Scheme of the paw depicting the nine subareas, in which nociceptive mechanical threshold was obtained, using calibrated pinprick (see Materials and Methods). The examined areas are proximal medial (HeelIn, HI), proximal middle (HeelM, HM) and proximal lateral (HeelOut, HO); median medial (MIn, MI), median middle (MM) and median lateral (MOut, MO); distal medial (FingersIn, FI), distal middle (FingersM, FM) and distal lateral (FingersOut, FO). (**B**) Changes in the nociceptive threshold measured using calibrated pinprick at the indicated time points after SNI (*red circles*) and sham surgery (white squares) at each of the examined areas (shown in *A*). Each subpanel demonstrates the changes in the nociceptive threshold measured in one of the subareas shown in *A.* The subpanels are organized and labeled according to the examined area (abbreviations and a red dot on the inset). *** *p* < 0.001; ** *p* < 0.01; * *p* < 0.05; ns—not significant, n = 6 rats in each group, RM two-way ANOVA). The baseline value represents the mean value of the three tests performed at three consecutive days before SNI or sham surgery. All of the values were normalized to the baseline value. If animals did not respond to the stimulus higher than 2× baseline value (120 gr, cut off), the area was considered to be denervated. Note that some areas became denervated after SNI, and all areas developed pain hypersensitivity. Note also that the duration of denervation and the onset of pain hypersensitivity vary among the nine areas. (**C**) Schematic representation of denervated areas after SNI. Note, the T-shaped areas which include the middle part of the paw as well as the distal areas closed to the fingers.

We based the DRG labeling approach on a method we previously described [17]. We used a pulled glass pipette attached to a syringe to inject the paw skin. At each injection site, ~0.5 µL of 10 mg/mL wheat germ agglutinin (WGA) conjugated to Alexa flour (AF, Invitrogen, Carlsbad, CA, USA) 488, 555, 594, or 647 was injected (Figure 1). Each color was subcutaneously injected by applying hand pressure onto a different area of the paw (Figure 1), namely—lateral middle area (MOut, MO), medial middle area (MIn, MI), median middle area (MM), and distal middle area (FingerM, FM, Figure 1). The MM and FM are the areas that undergo denervation following SNI, as shown in Figure 2C. The injections were performed at different time points in accordance with the behavioral tests (see below). We previously showed that vesicles containing the dye are transported back to the soma, eventually entering larger organelles in the lysosomal pathway and then get degraded within a week [17]. Therefore, we did all imaging between 30–40 h post target injection. At this time, the animals were re-anesthetized and decapitated. The L_3_-L_6_ DRGs (which innervate the paw) and the labeled skin paw were extirpated and fixed by immersing for 30 min. into paraformaldehyde and then washed with phosphate-buffered saline (PBS).

### 2.4. Image Acquisition

The samples were placed on slides containing vecta-shield and examined using a Zeiss LSM710 confocal microscope. For DRG imaging, we used a 25× oil objective with an NA of 0.8. For the skin, we used a 10× air objective with an NA of 0.4. We used a 488-nm argon laser to excite AF488 (490 to 523-nm emission); a 561-nm photodiode laser (562 to 584-nm emission) was used for AF555; a 594-nm photodiode laser (597 to 632-nm emission) for AF 594; and, a 633-nm photodiode laser (659 to 740-nm emission) for AF647. The gain was tuned to the point where the brightest dyed cells were not saturated among the four L_3_-L_6_ of the DRG somata images, for each channel. Each channel was separately imaged in a sequential manner.

### 2.5. Image Analysis 

The confocal images of the paw and the isolated DRG of each experimental animal were analyzed using FIJI software using the public domain NIH Image program (developed at the U.S. National Institutes of Health and available on the Internet at http://rsb.info.nih.gov/nih-image/). Each image was a merge of the four channels that were taken using confocal microscopy (see above). Each channel, which represents one of the injected dyes, was detected by different suitable excitation and emission filters. We used a two-pixel radius Gaussian filter applied to the target DRG confocal images and a one-pixel radius filter on the paw confocal images in order to reduce noise. We used the “FIJI analysis particles” plug-in to detect the somata, mark it, and measure its size, according to the predefined threshold. For the threshold measurement, each channel was separately analyzed, and the threshold was defined according to the channel maximal fluorescence level. The fluorescent spots that were smaller than the diffraction limit (occupied less than 150 µm^2^) were considered as noise and excluded from the analysis. Post hoc manual examination of the perimeter surrounding soma, and changes were made if needed.

We then examined the colors (AF 488—green, AF 555—blue, AF 594—red, AF 647—yellow), which we detected in the DRG images and related them to the colors in the injection sites of the paw. Given that the WGA-based dyes are uptaken by the axons from the periphery and retrogradely transported to the cell bodies [17] in the DRG, we could correlate between the specific cell body in the DRG to its peripheral innervation area in the paw. In all experiments, the detection of the somata and the paw was blindly performed with respect to the somata’s original color. The number of cells that innervate different paw territory was calculated. Subsequently, the somata diameter was measured to distinguish between nociceptive and non-nociceptive neurons.

### 2.6. Behavioral Analysis 

The animals were habituated to the testing environment for at least three sessions, after which baseline testing was performed. The room temperature and humidity remained stable during the entire experiment, and food and water were regularly provided.

Nociceptive threshold measurements were used to map the denervation areas and study the dynamics of the denervation and reinnervation. The nociceptive threshold was tested using calibrated pinprick (CPP), a system that we have developed. The paw was divided into nine separate areas—proximal medial (HeelIn, HI), proximal middle (HeelM, HM), and proximal lateral (HeelOut, HO); median medial (MIn, MI), median middle (MM), and median lateral (MOut, MO); distal medial (FingersIn, FI), distal middle (FingersM, FM), and distal lateral (FingersOut, FO, Figure 2). Calibrated pinprick (CPP) was made by attaching a pin tip to a von Frey filaments; hence, each pin-filament complex represented a calibrated pressure by which the pin is applied. The rats were wrapped in a cloth to which they were habituated to create a familiar environment and calm the rats during testing. Once the rat was held in place, they were held in the prone position. We pulled the left leg from under the cloth and held the leg delicately just under the paw (so as not to interrupt the paws motion). We then applied a variety of CPPs to each for the abovementioned nine areas, and Dixon’s up-down method (see above), in intervals of 3–4 pressings of each filament, was used to determine the nociceptive threshold. The pain threshold was defined by paw withdrawal, paw shake, or vocalization. The mean value of the stimulus, which evoked the response in control rats, was 61.7 ± 1.4 g (n = 50 rats). The value of 120 g was set as a cutoff value. The time of loss of sensation was determined as the first day in which the rat did not respond to the cutoff stimuli of 120 g. The time of the return of sensation was determined as the first day in which the rat responded to the 120 g or lower stimulus. The time of return to baseline levels was determined as the first day in which the rat response was similar to the baseline stimulus (60 g). The time of developing neuropathic pain was determined as the first day on which the rat responded to a stimulus of ≤15 g.

Mechanical allodynia was tested while using von Frey filaments (VF filaments). Each animal was placed in an elevated plastic chamber with a mesh floor. We gently pressed a VF filaments of different thicknesses against the plantar surface of the left paw until it bent. The VF filament was vertically pressed to the MM and FM areas (see above) of the plantar skin. The withdrawal threshold was determined based upon Dixon’s up-down method (every time a rat reacted to a certain hair stiffness, we decreased the stiffness until there was no more reaction) in intervals of 3–4 pressings for each hair. If the rat raised its paw for three or more times out of 10 applications, we considered it to be a positive response.

Mechanical hyperalgesia was tested using an adaptation of the pinprick test. To assure that we apply constant pressure of the pin to different animals and among different trials, we attached the pin tip (used in a conventional pinprick test) to a 60-g VF, and then pressed it vertically onto the plantar surface of the paw until the hair bent, and measured the duration of the resulted paw lifting. The test was performed in the same area the mechanical allodynia was examined (area at the center of the paw corresponding to MM and FM areas). We measured the response duration (paw lifting) by calculating the time from paw rising until the paw touched the floor again. We normalized all of the responses to the short paw lift response following the application of the calibrated pinprick to the uninjured animals, which was of 1 s duration.

### 2.7. Groups and Design of the Experiment

The experiment consisted of several sham and experimental groups. In the first sham group, rats (six animals) underwent the entire SNI procedure, but without the ligation and axotomy of the nerves. These animals were tested for mechanical hyperalgesia and allodynia for three months, after which their paws were injected with the dyes and their DRGs and paw skin removed. In some experiments, rats underwent sham surgery, as explained above. Their paws were injected with the WGA conjugated fluorophores one day after surgery, and then DRGs and paw skin were harvested. In the experimental groups, the rats underwent SNI surgery, and then rat’s DRGs and paw skin were removed according to a different set of behavioral criteria, which corresponded to the different stages following SNI injury (6 rats in each group): day 1—denervation; day 7—a return of sensation in some animals, day 14—a return of the sensation in all animals, days 21–28—development of allodynia and hyperalgesia; days 63–70 when neuropathic pain symptoms start to decrease and day 90—return to baseline (Figure 2).

### 2.8. Ouabain Experiments

In some experiments, we treated rats with ouabain (3.6 μg/kg, i.p.) following SNI and compared the innervation patterns and development of neuropathic pain symptoms to the untreated rats (treated with saline, 0.9% NaCl, B. Braun, Melsungen AG, Germany), which underwent SNI. For the behavioral experiments, ouabain (n = 7 rats) or saline (n = 5 rats) were injected i.p. immediately after SNI surgery and then given daily until the end of the experiments on day 30 after SNI. For the innervation experiments, the tissues were collected 18 days after SNI. Ouabain stock solution (1 mM, Sigma -Aldrich Inc., Rehovot, Israel) was prepared in saline (0.9% NaCl, B. Braun, Melsungen AG, Germany).

### 2.9. Measurement of Endogenous Ouabain Concentration

To measure whether the treatment with the 3.6 μg/kg ouabain was sufficient to increase ouabain concentration in the rats, we have measured endogenous ouabain concentrations. The procedure was performed, as previously described [18]. Rat’s blood was collected using retro-orbital bleeding, and then plasma samples were centrifuged (5 min., 15,000× *g*). The supernatant was first separated from high molecular-weight compounds, while using a 3000 NMWL membrane centrifugal filter. The lower molecular weight fraction (<3000) containing free steroids was diluted (1:1, *v*/*v*) with 0.1% trifluoroacetic acid (TFA). Each sample was loaded onto a Sep-pak C-18 column, which was then washed with 10 mL water containing 0.1% TFA, and the bound steroid was eluted with 80% acetonitrile. The solvent was evaporated and the residue dissolved in PBS. The amount of endogenous ouabain was measured by a sensitive, competitive inhibition ELISA [18]. The treatment with the 3.6 μg/kg ouabain significantly increased the ouabain concentration in blood (data not shown).

### 2.10. Experimental Design and Statistical Analysis 

The data are shown as mean ± S.E.M. or S.D. Differences between groups were analyzed using a two-tailed Student’s *t*-test or one/two-way ANOVA analysis of variance, followed by Sidak or Bonferroni post hoc tests or chi-square independence test when appropriate. The criterion for statistical significance was *p* < 0.05.

Sample size calculation: For in vivo experiments, we collected and analyzed all of the data using a minimum of five animals per group.

The number of replicates (n) for each experiment is given either in the Figure legends or in the Results. If a representative example is shown, we always explain how representative it is, i.e., how many cells/animals showed a similar effect. The inclusion criteria for each experiment are described in the Materials and Methods section, above.

## 3. Results

### 3.1. Painbow, a Method to Characterize Reinnervation Following Nerve Injury

In this study, we aimed to identify the DRG neurons whose axons sprout to the denervated areas of the paw in the model of SNI. We adapted a multispectral retrograde labeling Neuronal Positioning System (NPS) technique that we previously described [17], to correlate the axonal projections at the skin to somata of DRG neurons. The NPS approach allows for the identification of neuronal somata innervating specific tissue areas. This approach is based on the injection to a target organ of multiple spectrally separated fluorescent dyes that were conjugated to Wheat Germ Agglutinin (WGA), which were retrogradely transported to cell somata by the axons innervating the target organs. Spectral analysis of combinations of these dyes at the cell bodies permits the mapping of axonal projections of many axons simultaneously [17]. Here, we used this approach to identify cells that innervate specific areas in the skin of rat hind paw and study changes in innervation patterns following spared nerve injury (SNI) [14]. Accordingly, we injected four spectrally separated fluorophores that were conjugated to WGA (AF 488—green, AF 555—blue, AF 594—red, AF 647—yellow, see Materials and Methods) to specific areas of the paw (Figure 1A).

The examination of the paw skin using confocal microscopy verified that the dyes remained in the paw 40 h post-injection (Figure 1A, right inset). For each examined animal, we determined the diffusion pattern of each dye (see, for example, Figure 1A, right inset), enabling the correlation between the skin area and labeled DRG somata (Figure 1A, left inset, Figure 1B). The DRGs that innervated these areas were then excised, and confocal images were performed to analyze the labeling of DRG somata. This procedure was performed to L_3_-L_6_ DRGs known to innervate the paw skin (Figure 1, see Figure 1A, left inset, for example of L_5_ DRG). Images of L_3_-L_6_ DRGs revealed multiple DRG somata labeled by different dyes (Figure 1A, left inset, Figure 1B). All four dyes injected into the paw were detected in DRG somata of different lumbar levels (Figure 1B,C), implying that these dyes were transported retrogradely into DRGs somata from the injected area of the paw. We then counted the number of cells that were labeled with a specific dye and correlated this number with the paw skin area of the corresponding dye, i.e., the number of red (AF594), green (AF488), yellow (AF647), and blue (AF555)—labeled cells innervating red (AF594), green (AF488), yellow (AF647), and blue (AF555) labeled area in the paw, respectively (Figure 1B,C). To control for a possible difference in dyes distribution, we performed a series of experiments, where we injected the same combinations of four dyes into the same areas of the paw, but in a different order. We found that the number of cells labeled with the dye corresponding to a specific skin area remained the same (149 ± 15 neurons, *p* = 0.98, one sample *t*-test compared to the mean value, n = 5 rats). We also tested for the reproducibility of this technique by injecting the same amount of dye to a specific area and counting the labeled somata. We found that the number of labeled somata was not different between different injections (290 ± 25 neurons, *p* = 0.98, one sample *t*-test as compared to the mean value, n = 5 rats). The above control experiments suggest that this technique can be used in a semi-quantitive manner to identify and estimate the amount of DRG neurons innervating distinct skin areas. Given the multispectral nature of this technique, we named it Painbow.

For all of the following experiments, the injection of the fluorophores that were conjugated to WGA remained consistent: blue, AF555 dye was injected into the centro-medial area of the paw (Figure 1A), which is mainly innervated by the saphenous nerve [7,8,14]. Green AF488 and yellow AF647 dyes were injected into the centro-median area of the paw (Figure 1A), being mainly innervated by the tibial nerve [7,8,14] and red AF594 dye was injected into the centro-lateral areas (Figure 1A), which are mainly innervated by the sural nerve [7,8,14]. The calculation of the number of cells labeled with a specific dye throughout the examined DRG segments shows that AF555 was mainly found in L_3_ DRG (43 ± 9.9% of all labeled cells in L_3_ were labeled with AF555, n = 5 rats, Figure 1B,C). AF488 and AF647 were mainly found in L4 and L5 segments, respectively (49.4 ± 5.5% of all labeled cells in L_4_ were labeled with AF488, 56.34 ± 9.2% of all labeled cells in L_5_ were labeled with AF647, n = 5 rats, Figure 1B,C). AF594 was predominantly found in L_6_ DRG (71.5 ± 5.7%, n = 5 rats, Figure 1B,C). These results show that Painbow accurately captures the relation between skin areas and the innervation segment.

Finally, we examined whether Painbow can be used to study changes in innervation patterns during denervation and reinnervation. First, we mapped in detail the areas of the paw which undergo denervation following SNI. Previously, it was demonstrated, while using von Frey filaments (VF), that SNI leads to denervation of the middle plantar area of the paw [7,8,14]. We measured the nociceptive mechanical threshold utilizing a calibrated pinprick approach (CPP, see Materials and Methods) to map the denervation areas and study the dynamics of the denervation and reinnervation of the paw following SNI (Figure 2). To that end, we divided the plantar area of the paw into nine sub-areas (3 × 3, Figure 2A): proximal medial (HeelIn, HI), proximal middle (HeelM, HM) and proximal lateral (HeelOut, HO); median medial (MIn, MI), median middle (MM), and median lateral (MOut, MO); distal medial (FingersIn, FI), distal middle (FingersM, FM), and distal lateral (FingersOut, FO). We then applied the pin tip that was attached to a VF filaments of different diameters onto each of these areas to define for each of them the exact force (instead of response duration as in regular pinprick) in which the noxious stimuli (pin) led to a response. It allowed for unifying noxious mechanical stimuli among different animals and throughout the experiment. The paw division, together with the small size of the pin tip, permitted the reliable mapping of the responses to these unified stimuli applied to the defined paw areas. Before SNI surgery or in sham-operated animals, all of the animals responded by paw lifting, paw shaking, or vocalization only when 60 g VF pin was applied. We defined this as threshold and normalized all the responses to this value (see Materials and Methods, Figure 2). If animals did not respond to the force equivalent to 2× the threshold (120 g CPP, cut off), we considered this area as denervated area. The end of the denervation period was defined by the time animals began responding to a CPP of 120 g and below. The application of CPP onto paws after SNI showed that SNI produces a T-shaped denervation area (Figure 2C). This denervation area occupied the middle third (HM, MM, and FM areas, see above and also Materials and Methods) of the paw as well as a distal third (closed to the finger) of the paw (FI and FO areas, Figure 2C). The denervation at these areas was detected from day one after SNI and lasted for about four weeks in the FM area, three weeks in MM area, two weeks in the FO area, one week in the FI area, and three days in HM area (Figure 2B). All other areas (MI, HI, MO, HO) responded to CPP of 60 g and below during the whole period following SNI and, therefore, were not considered to be denervated.

The development of SNI-induced mechanical hyperalgesia (measured as a decrease in nociceptive threshold) was observed in all nine areas but developed at different time points after the SNI procedure (Figure 2B). In areas that underwent denervation, mechanical hyperalgesia started shortly (within about a week) after sensation had returned to baseline (Figure 2B). In the areas that did not undergo denervation, the onset of hyperalgesia varied between one week after SNI in HO, two weeks in HI, three weeks in MI, and four weeks in MO (Figure 2B).

**Figure 3:**
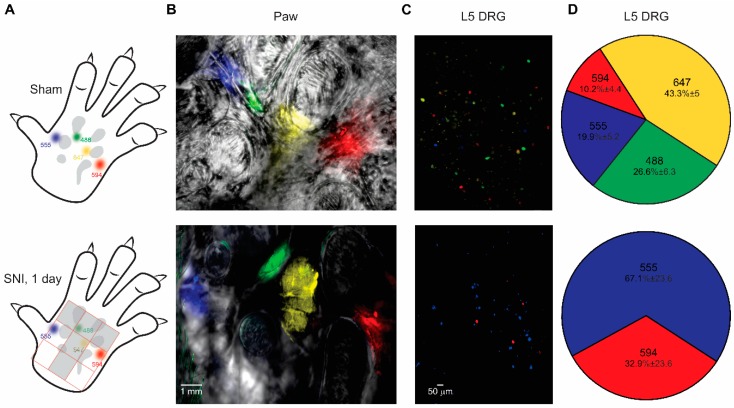
The Painbow detects changes in innervation. (**A**) Scheme representing the injection arrangement. The four dyes were injected into the paw one day after either sham (upper panel) or SNI (lower panel) surgery. The dyes were injected to sham and SNI groups in a similar arrangement and in accordance with the denervation pattern (depicted in the lower panel, as a grey “T-shaped” area), such that AF555 and AF594 were injected into medial and lateral areas (respectively) of the paw that does not undergo denervation. AF488 and AF647 were injected into the areas which were denervated after SNI. (**B**) Representative fluorescent images of the skin demonstrating the arrangement of the injections one day after sham surgery (upper panel) and 1 day after SNI (lower panel). A representative of six rats for sham and SNI groups (**C**). Representative fluorescent images of L_5_ DRGs from sham-operated (upper panel) and SNI (lower panel) rats, removed 40 h after dyes were injected into the skin. Note that in DRG from a sham-operated animal, all colors are present, whereas in DRG from the animal after SNI, only colors injected into areas that are not underwent denervation (AF555 (blue) and AF594 (red)) are present. A representative of 6 rats for sham and SNI groups. (**D**) Distribution of dyes in L_5_ DRGs from sham-operated animals (upper panel) and rat after SNI (lower panel). Note that in L_5_ DRG from sham-operated rats the majority of the cells were labeled with AF647, but all colors were present, suggesting that L_5_ DRG neurons send their axons into all labeled skin areas shown in *A*. In animals after SNI no cells labeled with the dyes injected into the denervated areas were found, n = 6 rats in each group.

**Figure 4:**
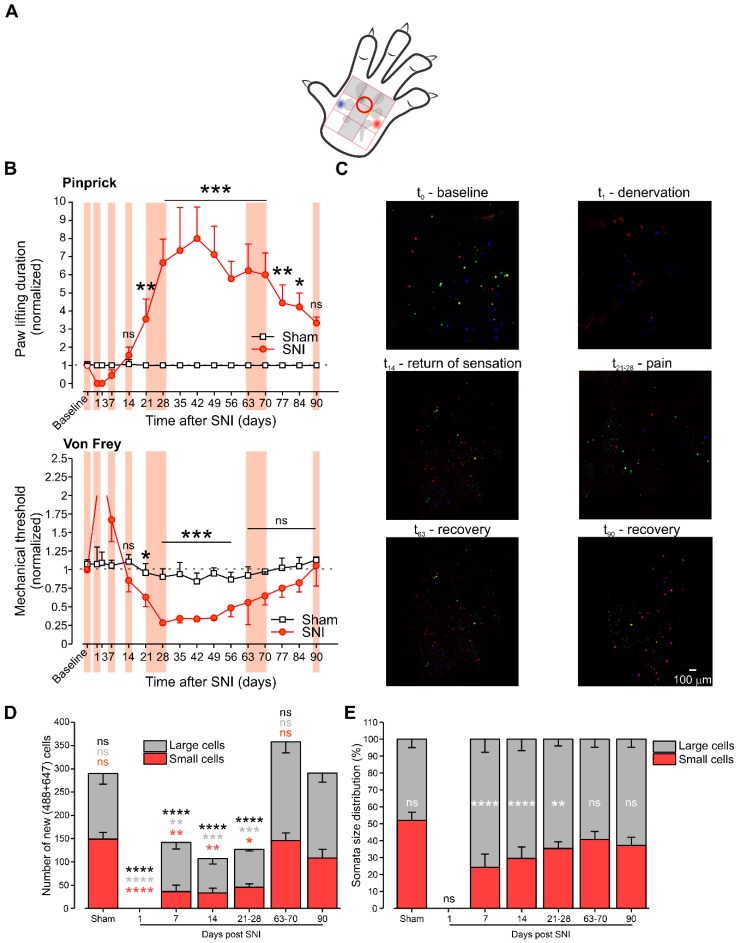
During the development of neuropathic pain symptoms, the skin is reinnervated by a lower number of neurons and primarily by non-nociceptive neurons. (**A**) Scheme depicting the area (red circle) to which calibrated pinprick and von Frey (VF) were applied to examine the responsiveness of the rats to noxious and innocuous mechanical stimuli, respectively. Note that the chosen area represents the part of the paw skin, which underwent denervation. (**B**) Changes of the response to pinprick (upper panel) and VF (lower panel) at the indicated times following sham surgery (white squares) and SNI surgery (red circles). The baseline value represents the mean value of the 3 tests performed at three consecutive days before SNI or sham surgery. All of the values were normalized to the baseline value. *** *p* < 0.001; ** *p* < 0.01; * *p* < 0.05; ns—not significant, n = 6 rats in each group, RM two-way ANOVA). Pink vertical areas outline the times at which some of the animals were pulled out of the experiments, and their paws were injected with the AF dyes. These times correspond to the following stages: 1 day after SNI - denervation stage - where no animals responded to the cut off value of the VF (2 X baseline value), and no animals responded to pinprick; seven days after SNI—recovery of sensation in half of the examined animals; 14 days after SNI—return to baseline (recovery of the sensation in all animals); 21–28 days after SNI—development of hyperalgesia and allodynia; 63–70 days after SNI—decrease in hyperalgesia and allodynia; 90 days after SNI—full recovery from hyperalgesia and allodynia. (**C**) Representative (of six rats) fluorescent photomicrographs of L_5_ DRGs extirpated 40 h after paw injection of dyes at the indicated times after SNI, corresponding to the abovementioned behavioral stages. (**D**) Changes in the number of L_3-6_ DRG somata labeled with either AF488 or AF647 (injected into denervated areas) at the denervation stage (day 1 after SNI), recovery of the sensation stage (7 and 14 days after SNI), development of the hyperalgesia and allodynia stage (21–28 days after SNI); decrease in hyperalgesia and allodynia (63–70 days after SNI) and full recovery from hyperalgesia and allodynia (90 days after SNI), plotted against the number of L_3-6_ DRG somata labeled with either AF488 or AF647 in animals 90 days after sham surgery. Each bar is composed of the number of small (nociceptive, red) and large (non-nociceptive cells, grey) cells. Comparison is with the number of cells at day 90 after SNI. Black letters and asterisk—comparison between a total number of cells at different time points; red letters and asterisk—comparison between the number of small cells at different time points; grey letters and asterisk—comparison between the number of large cells at different time points. **** *p* < 0.0001; *** *p* < 0.001; ** *p* < 0.01; * *p* < 0.05; ns—not significant, n = 6 L_3-6_ DRGs in each group, two-way ANOVA. (**E**) Bar graph depicting the distribution (in %) of large (grey) and small (red) L_3-6_ DRG neurons labeled with AF488 or AF647 at the indicated time points and plotted against the L_3-6_ DRG neurons size distribution from animals 90 days after sham surgery. Comparison is with the number of cells at day 90 after SNI. **** *p* < 0.0001; ** *p* < 0.01; ns—not significant, n = 6 L_3-6_ DRGs in each group, two-way ANOVA, comparisons are between the large and small cells at each time point.

The detailed mapping of innervated and denervated areas gave means to further validate the Painbow approach and examine if it accurately captures changes in innervation following nerve injury. If Painbow indeed reflects the innervation patterns, the injection of the dyes into denervated areas MM and FM should not produce any labeling of the DRG neurons, whereas the dyes injected into innervated MI and MO areas should be present in the DRG neurons. We injected AF555 and AF594 dyes into MI and MO areas, which do not undergo denervation (Figure 2C), and AF488 and AF647 into denervated MM and FM areas of the middle paw (Figure 2C; Figure 3A,B) in order to examine this hypothesis. The dye injections were performed one day after either sham or SNI surgery. Forty hours after the injection, we analyzed the L_3-6_ DRGs’ labeling and compared the labeling after SNI to the labeling after sham surgery (Figure 3). Our results, shown above (Figure 1), suggest that L_5_ DRG innervates all of the injected areas. Indeed, in sham-operated animals, L_5_ DRG somata were labeled with all injected dyes (Figure 3C,D, upper panels). Importantly, after SNI, only AF555 and AF594 dyes (which were injected into areas that do not undergo denervation) were present in L_5_ DRG somata (Figure 3C,D, lower panels). Similarly, no AF488 and AF647 were detected in all examined segments (Figure 4D), confirming a lack of dye transport and hence an absence of DRG axons in denervated areas. These results show that dyes indeed are transported retrogradely from the injected skin into DRGs and that the presence of specific dyes in the DRG requires an intact innervation pathway.

All of the above suggests that Painbow can be used to map the axonal projections of DRGs somata. It also provides a platform to examine the changes in the innervation patterns during the development of neuropathic pain in denervated areas.

### 3.2. Painbow Reveals Changes in Innervation Patterns Following SNI-Induced Denervation, Which Correlate with the Development of Neuropathic Pain

Previous studies suggested that changes in the innervation pattern during the reinnervation of denervated areas following neuropathic injury contribute to the development of neuropathic pain [7,8,9,13]. The effect of the reinnervation on the development of neuropathic pain could result from either change in the overall number of fibers innervating the area, or changes in the types of cells innervating the area or both. We examined the development of mechanical sensation at denervated areas of the paw and correlated it with the number and size distribution of DRG somata reinnervating these areas to study the number and profile of DRG neurons reinnervating specific denervated skin areas following SNI. We first measured the development of neuropathic pain symptoms—mechanical hyperalgesia (using pinprick) and mechanical allodynia (using VF filaments) at the area, which underwent denervation following SNI (marked by a red circle, Figure 4A). The first three days after SNI, the animals did not respond to the application of pinprick connected to a 60 g VF filament applied to the examined area (Figure 4B, upper panel), implying that the examined areas underwent denervation. Seven days after SNI, three out of six animals did not respond to the pinprick application, the other three rats responded by paw lifting with a duration similar to the baseline duration (the duration measured before the surgery). Fourteen days after surgery, all of the examined animals returned to the baseline values (Figure 4B, upper panel), suggesting reinnervation 14 days after SNI. Measurement of mechanical threshold using VF filaments showed a similar time course of denervation and reinnervation: lack of response 3 days after SNI, and return to baseline two weeks after SNI, (Figure 4B, lower panel). Importantly, three weeks after SNI, all of the examined animals responded to the application of calibrated pinprick with a significantly longer paw lifting duration. This mechanical hyperalgesia continued until 12 weeks after SNI and then decreased to baseline levels three months after SNI. The onset of mechanical allodynia was similar to the development of mechanical hyperalgesia, but the duration was shorter (about nine weeks, Figure 4B, upper panel).

In parallel with monitoring of the changes in mechanical thresholds, we injected AF555 and AF594 into non-denervated areas—MI and MO, and AF488 and AF647 into denervated areas—MM and FM. We assumed that DRG neurons labeled with AF488 and AF647 at later time points after SNI (when sensation returns or pain develops) reflects DRG neurons, which reinnervate these denervated areas, since we showed that shortly after SNI neither AF488 nor AF647 are present in the DRGs (Figure 3 and Figure 4C for L_5_; Figure 4D for all examined DRGs). Accordingly, we injected AF488 and AF647 at different time points following SNI, in correlation with the development of denervation, reinnervation, mechanical hyperalgesia, and allodynia. We examined the number of AF488 and AF647 labeled L_3-6_ DRG neurons at the following time points (Figure 4B,C): denervation—one day after SNI; recovery of sensation in half of the examined animals—seven days after SNI; return to the baseline (recovery of the sensation in all animals)—14 days after SNI; development of hyperalgesia and allodynia—21–28 days after SNI; decrease in hyperalgesia and allodynia—63–70 days after SNI, full recovery from hyperalgesia and allodynia—90 days after SNI. We compared the number and type of labeled cells at each time point to the number and type of AF488 or AF647 labeled cells in DRGs excised from animals that were injected with the dyes 90 days after sham surgery. As expected, the return of sensation was accompanied by the appearance of cells that were labeled with AF488 and AF647 dyes injected into the denervated areas, suggesting that these cells reinnervate MM and FM. Interestingly, the number of cells innervating these areas changed following SNI, and this change was in correlation with the developing symptoms (Figure 4D). In sham-operated animals, we counted 290 ± 25 neurons in L_3-6_ labeled with either AF488 or AF647 (n = 5 rats, Figure 4D, left). No cells labeled with AF488 or AF647 were detected one day after SNI (Figure 4D), and no responses were evoked from these areas at this time point, as described above. Subsequently, on day 7, some animals (three out of six rats) started to respond to pinprick, and VF applied to this area. In those animals, a substantially smaller number of neurons (142 ± 20 neurons, n = 3 rats, Figure 4D, left) were labeled by either AF488 or AF647. When all of the animals responded to the pinprick and VF at day 14, the number of cells innervated initially denervated areas was still significantly lower than the number of cells innervating these areas in sham-operated animals (107 ± 19 neurons, n = 5 rats, *p* < 0.0001, two-way ANOVA, Figure 4D, left). Interestingly, at the time of development of neuropathic pain, when animals responded with abnormally high sensitivity to innocuous and noxious mechanical stimuli, the number of cells that were labeled with AF488 or AF647 remained significantly lower than in sham-operated animals or in animals recovered from neuropathic pain symptoms (126 ± 7 neurons, n = 5 rats, *p* < 0.0001, two-way ANOVA, Figure 4D, left). Finally, two months after SNI, when mechanical allodynia was abolished, and mechanical hyperalgesia was alleviated, the number of cells labeled with AF488 or AF647 became similar to the number of cells in sham-operated animals (358 ± 20 neurons, n = 5 rats, *p* = 0.19, two-way ANOVA, Figure 4D, left).

We also analyzed the ratio between small (about 25 μm, nociceptive) and large (more than 30 μm, non-nociceptive) AF488 and AF647 labeled L_3-6_ DRG neurons at the same time points, as described above. Surprisingly, the ratio of nociceptive and non-nociceptive cells innervating the MM and FM areas also changed after denervation. In sham-operated animals, the MM and FM areas were innervated by a similar amount of nociceptive and non-nociceptive cells (Figure 4D,E). However, in SNI-operated animals, during the return of sensation (days 7 and 14) and at the hyperalgesia and allodynia stage (days 21–28), the MM and FM areas were mostly innervated by large, non-nociceptive neurons (about 70–80% of neurons, Figure 4D,E). At the time when mechanical allodynia was abolished, and mechanical hyperalgesia was diminished (days 63–70), this area was innervated by a similar number of nociceptive and non-nociceptive neurons, such that the ratio was similar to that of the sham-operated animals (Figure 4D,E).

All of the above demonstrates that, after SNI, at the time points when denervated areas develop neuropathic pain symptoms, such as mechanical allodynia and hyperalgesia, these areas were reinnervated by a significantly lower number of DRG neurons. It also shows that, at this time period, the majority of cells that innervate these areas are large, presumably, non-nociceptive neurons. The decrease in neuropathic pain was accompanied by increased innervation by DRG and the restoration of the equilibrium between the contribution of nociceptive and non-nociceptive neurons.

**Figure 5:**
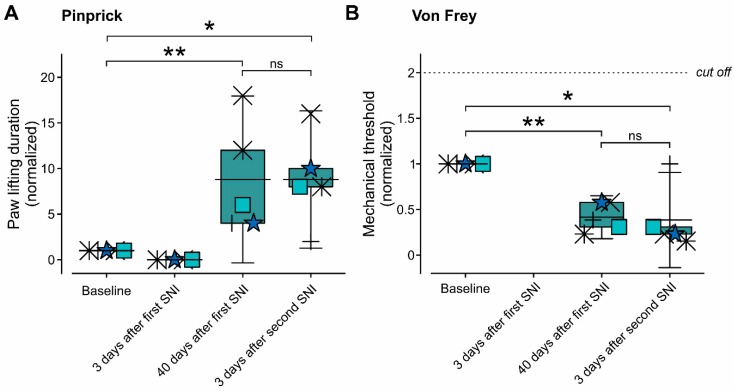
Reinnervation does not originate from lacerated tibial and peroneal nerves but is resulted from sprouting from the axons innervating neighboring areas. (**A**,**B**) Box plots and paired individual values of normalized paw lifting latencies, measured using calibrated pinprick (**A**) and mechanical threshold, measured using VF (**B**) before SNI surgery (baseline); three and 40 days after the first SNI and three days after repeated SNI. Note no values for the mechanical threshold 3 days after SNI (**B**) as all animals did not respond to the cut off value of the VF. Note no significant difference between the values of the mechanical threshold and paw lifting duration measured 40 days after SNI and three days after the second SNI. The baseline value represents the mean values of three tests performed at three consecutive days before SNI or sham surgery. All of the values were normalized to the baseline value. ** *p* < 0.01; * *p* < 0.05; ns—not significant, RM one-way ANOVA. Box plots presented in the figures depict mean; 25th; 75th percentile and SD.

The axons that reinnervate the denervated areas could originate from sprouting of uninjured axons of sural or saphenous nerves [11,12]. Alternatively, they could regenerate from the cut axons of tibial and peroneal nerves [6]. If the latter is the case, a second SNI, performed 3–5 weeks after the first SNI, would abolish the mechanical allodynia observed at this stage, as it will again produce denervation. Accordingly, we examined whether a second SNI, performed after the first SNI-induced mechanical hyperalgesia and allodynia are fully developed, will affect the changes in the mechanical threshold that was induced by the first SNI. Similar to the results that are demonstrated in Figure 2 and Figure 3, three days after the first SNI, the application of either pinprick or 120 g VF filament did not evoke a response. Forty days after the first SNI, prominent mechanical allodynia and hyperalgesia were observed (Figure 5A,B). The second SNI surgery that was performed at this stage did not change the levels of mechanical allodynia and hyperalgesia (Figure 5A,B), which suggested that the neurons that convey this pain hypersensitivity are not from axons of tibial and peroneal nerves, but rather sprouting from neighboring uninjured fibers of sural or saphenous nerves.

### 3.3. Ouabain Promotes Reinnervation and Diminishes Neuropathic Pain

**Figure 6:**
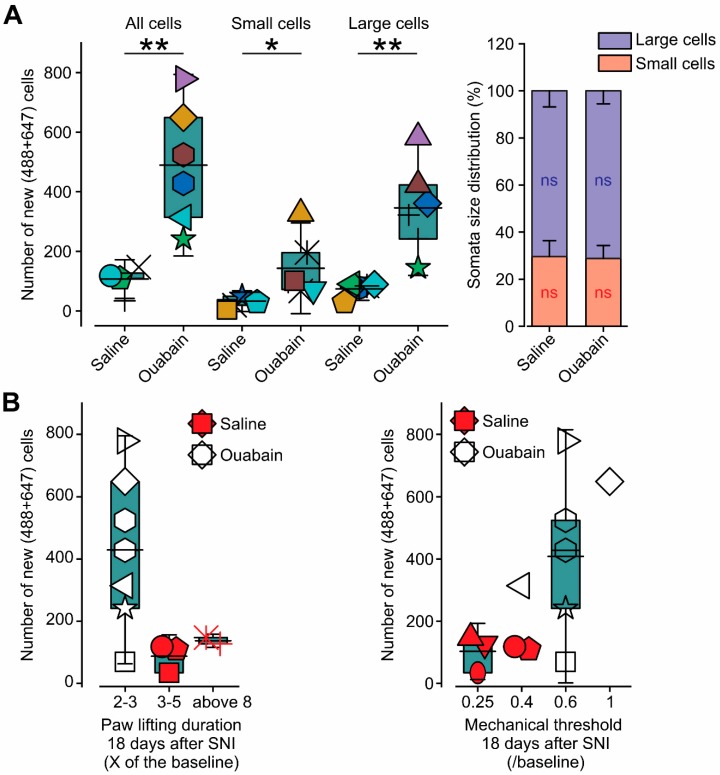
Ouabain promotes reinnervation of the denervated skin areas. (**A**) Left, Box plots and individual values of the total number of L_3-6_ DRG somata labeled with AF488 or AF647; a number of small cells and number of large cells labeled with AF488 or AF647, 18 days after SNI in animals treated with ouabain and saline. ** *p* < 0.01; * *p* < 0.05; ns—not significant, Student *t*-test. Box plots presented in the figures depict mean; 25th; 75th percentile and SD. Right, bar graph depicting the distribution (in %) of large (lilac) and small (pink) DRG neurons labeled with AF488 or AF647, 30 days after SNI in animals treated with ouabain and saline (shown in **A**, left), ns—not significant, comparisons are between the number of cells in animals treated by ouabain and saline. (**B**) Box plots and individual values of the number of DRG somata labeled with AF488 or AF647 in the DRGs extirpated from rats treated with either saline (red symbols) or ouabain (white symbols) 18 days after SNI and plotted according to the level of mechanical hyperalgesia (measured as the duration of paw lifting following pinprick and presented as fold change from the baseline value, left) and mechanical allodynia (measured as VF mechanical threshold and normalized to the baseline value, right). Box plots presented in the figures depict mean; 25th; 75th percentile and SD.

Our data show that the development of allodynia and hyperalgesia is accompanied by a decrease in the number of DRG neurons reinnervating the denervated skin, and vice versa; a reduction in the levels of allodynia and hyperalgesia parallels the increase in the number of neurons innervating the denervated area (see Figure 4D). We also demonstrated the dis-equilibrium between large and small DRG neurons’ innervation during the development of neuropathic pain. These results suggest that the changes in the innervation patterns, namely a decrease in the number of innervating cells and an increase in the proportion of the large cells’ innervation, could contribute to the development of neuropathic pain. If the decrease in innervation indeed contributes to neuropathic pain, the enhancement of innervation should facilitate the alleviation of neuropathic pain symptoms. We used ouabain in order to examine this hypothesis, which, when applied in low doses, is known to facilitate neuronal proliferation after injury [19] and promote neurite growth [20]. Indeed, in animals that were treated with a low dose of ouabain (3.6 μg/kg, i.p., see Materials and Methods), daily after SNI, the number of cells innervating the MM and FM areas was significantly larger than in animals that were treated with saline (*p* < 0.01, n = 6 rats, Student *t*-test, Figure 6A, left). Treatment with ouabain led to a significant increase in nociceptive and non-nociceptive cell innervation (*p* < 0.01 for non-nociceptive cells; *p* < 0.05 for nociceptive cells, n = 6 rats, Student *t*-test, Figure 6A, left). However, the treatment with ouabain did not affect the ratio between the nociceptive and non-nociceptive cells, such that, on day 18 after SNI, the denervated areas were primarily innervated by non-nociceptive cells, similarly to untreated animals or animals that were treated with vehicle (Figure 6A, see also Figure 4D). Moreover, we examined the number of cells innervating the MM and FM in saline- and ouabain-treated animals and correlated it to the level of mechanical allodynia and hyperalgesia 18 days after SNI. The number of cells innervating MM and FM areas in animals treated with saline was 107 ± 19 neurons (n = 5 rats) and was similar to the number of neurons in untreated animals after SNI at this stage (Figure 4D). All of the saline-treated rats exhibited substantial mechanical allodynia and hyperalgesia (Figure 6B). All ouabain-treated animals showed a significantly higher number of cells innervating the examined areas (429 ± 92 neurons, Figure 6B, n = 7 rats, *p* < 0.01, Student *t*-test). Importantly, ouabain-treated animals showed substantially lower levels of mechanical allodynia and hyperalgesia (Figure 6B). These data show that, after SNI, ouabain enhances peripheral innervation into denervated areas, and this increased innervation is accompanied by a decrease in neuropathic pain.

**Figure 7:**
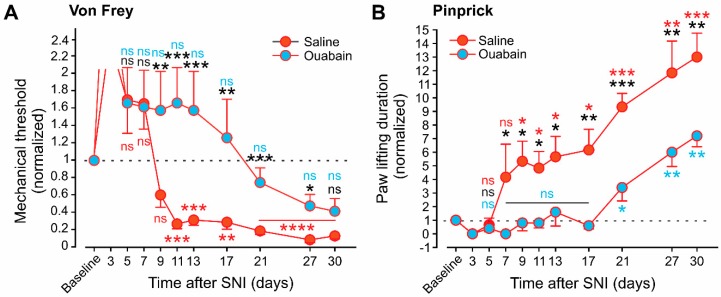
Ouabain diminished neuropathic pain. (**A**,**B**). Changes of the response to VF (**A**) and pinprick (**B**) at the indicated times following SNI in animals treated with ouabain (blue circles) and saline (red circles). The baseline value represents the mean value of the 3 tests performed at three consecutive days before SNI or sham surgery. All of the values were normalized to the baseline value. The dotted line indicated the baseline values. Black letters and asterisk—two-way RM ANOVA with posthoc Bonferroni comparison between saline and ouabain groups; blue letters and asterisk - one-way RM ANOVA with posthoc Bonferroni comparison between baseline values and values at different time points in the ouabain group; red letters and asterisk—one-way RM ANOVA with posthoc Bonferroni comparison between baseline value and values at different time points in the saline group. *** *p* < 0.001; ** *p* < 0.01; * *p* < 0.05; ns—not significant, n = 6 rats in each group.

We studied the development of neuropathic pain symptoms (mechanical allodynia and hyperalgesia) in ouabain-treated animals along the first 30 days after SNI and compared it to the saline-treated animals to further correlate between increased innervation and decrease in neuropathic pain. After the period of denervation, which was of similar duration in the two groups, saline-treated animals developed mechanical hyperalgesia (measured using pinprick) and allodynia (examined using VF filaments), which lasted for the whole examined period. Importantly, ouabain-treated animals did not develop mechanical allodynia (Figure 7A). Moreover, ouabain-treated animals did not demonstrate a significant increase in paw lifting duration during the first two and a half weeks after SNI (Figure 7B). Three weeks after SNI, ouabain-treated animals started to exhibit mechanical hyperalgesia, which was significantly milder than those in the saline-treated animals. Collectively, these results show that ouabain alleviates nerve-injury mediated neuropathic pain, probably by promoting reinnervation and sprouting into denervated areas.

## 4. Discussion

Chronic neuropathic pain is often a result of peripheral nerve injury. The aberrant activity of the injured nerves produces plastic changes at the PNS and CNS, initiating, and also maintaining, the pain. Additionally, it has been suggested that reinnervation of the denervated areas by virtue of its irregularity also contributes to the development of neuropathic pain. Here, we show that, indeed, the innervation pattern following denervation is different from the normal innervation pattern. We also show that the changes in the innervation pattern are correlated with the development of neuropathic pain and that the reversion of these changes alleviates the neuropathic pain symptoms.

We adopted a multi-spectral retrograde labeling approach, which we previously developed to map the location of many axons simultaneously, to characterize the innervation pattern in normal conditions and study how it changes following denervating nerve injury [17]. This approach, called the Neuronal Positioning System (NPS), is based on an innate axonal transport of spectrally separated fluorescent dyes conjugated to WGA and transported from the innervated area to the cell bodies by the axons innervating this area. We previously used this approach to define the axonal arborization area of individual ganglion cells into the salivary gland and map axonal projections between thalamus and cortex, by applying the dyes into the salivary gland and primary somatosensory cortex, respectively [17]. Here, we injected the dyes into distinct plantar areas of the rats’ hind paw. Similar to what we showed for parasympathetic and thalamocortical axons, here too, the dyes were uptaken by the peripheral sensory axons innervating the paw skin and retrogradely transported into corresponding DRG somata. We assumed that the color of the DRG somata corresponds to the skin area, which these DRG neurons innervate. Indeed, when we injected AF488 and AF647 dyes into denervated areas, no DRG somata were labeled with AF488 and AF647, and AF488 and AF647 appeared again only after the recovery of sensation. These results suggest a correlation between the color that was injected into the hind paw skin and the color of the DRG somata. Thus, by injecting multiple dyes into the paw skin, we identified the population of DRG neurons that innervate specific areas of the paw in normal conditions. By injecting the skin with the dyes and examining the distribution of DRG neurons at each stage after nerve injury: during the denervation, the reinnervation, and the development of neuropathic pain symptoms, we studied how the populations of DRG neurons that innervate specific areas of the paw are modified following nerve injury. We show that this approach could be useful in studying changes in innervation patterns during the development of neuropathic pain.

The majority of the current approaches studying changes in innervation following nerve injury use immunohistochemical analysis of paw skin biopsies to characterize changes in the density of specific markers against TrkA, P2X3, CGRP or NF-200 to examine peptidergic, non-peptidergic, and myelinated fibers, respectively [7,8,9,13]. These approaches allow for an analysis of the density of specific fibers in different skin layers, which show differential innervation and reinnervation patterns following nerve injury [7,8]. In the Painbow approach, dyes are injected subcutaneously, and, therefore, DRG neurons sending their axons both to epidermis and dermis will be labeled, and the identification and quantification of the innervating neurons occurs at the level of DRG somata and not fibers at the skin. Thus, it allows for an accurate estimation of change in the number of innervating cells and not just the extent of peripheral sprouting. Previous studies using reconstruction of paw skin following SNI demonstrated that, during the development of neuropathic pain, the density of Substance P containing peptidergic and P2X3 expressing non-peptidergic fibers is decreased [8], while the density of CGRP containing fibers is increased as compared to the normal levels, leading to the hyperinnervation of the initially denervated skin [7]. These results suggest that the initially denervated skin is reinnervated primarily by CGRP containing neurons. Our results show that the development of neuropathic symptoms is correlated with a decrease in the number of both small and large DRG neurons innervating previously denervated paw skin. The most probable explanation for this decrease is the lesion of the axons, which prevents the transport of AF488 and AF647 dyes from the examined areas to DRG somata. However, the apoptosis of the injured and neighboring DRG neurons following SNI-type nerve injury could be another explanation for the decrease in innervating DRG neurons [21,22]. The origin of the newly labeled DRG somata is most probably the result of the dye uptake by the axons, which sprouts from the neighboring areas and not from the regrowth of injured axons [7,11,12] (see also Figure 5, here). The decreased number of innervating somata, together with the increased number of CGRP-immunoreactive axons innervating denervated areas [7], suggest that each de-novo innervating DRG neuron increases the complexity of its terminal fibers. This increase in the terminal branching of individual DRG neurons might affect neuronal excitability [23,24,25,26] and contribute to the development of pain hypersensitivity from the initially denervated areas. Moreover, since all of these DRG neurons also innervate uninjured skin areas, an increase in their excitability, resulted from excessive branching, can also facilitate the development of increased pain sensitivity from the uninjured areas, which also develops following SNI [7,8,14] (see also Figure 2 here).

The ability of Painbow to identify and classify DRG neurons reinnervating injured areas is important in light of recent results, demonstrating that, in neuropathic rats, the expression of CGRP is reduced in small nociceptive neurons, but substantially increased in large non-nociceptive neurons [27]. These results imply that immunohistochemical identification of the reinnervating terminals in the skin biopsies might not be sufficient in predicting their effect on the development of neuropathic pain. Using Painbow, we discovered that the initially denervated areas are reinnervated by a substantially greater proportion of the large DRG neurons. These data are in correlation with a growing body of evidence showing the key involvement of large non-nociceptive neurons in the development of neuropathic pain [27,28]. Importantly, it has been shown that selective blockade of non-nociceptive peripheral neurons, prevents the development of neuropathic pain [28]. Selective blockade of nociceptive neurons blocks acute pain [28,29], but it has no effect on the development of mechanical allodynia [28]. These results suggest that non-nociceptive neurons are the source for the abnormal input, which drives the CNS and underlies the development of central sensitization. Given that these neurons overexpress CGRP in neuropathic pain conditions [27] and also that the amount of CGRP-immunoreactive neurons is increased following nerve injury [7], it could be that these non-nociceptive neurons are the neurons that primarily sprout into previously denervated areas. The characterization of the neurochemical properties of the large neurons, which innervate previously denervated areas, by combining Painbow with immunohistochemical analysis of the labeled neurons, could be useful for further understanding the role of different types of DRG neurons in reinnervation and, thus, their contribution to the development of neuropathic pain. We show that, although large DRG neurons provide the main innervation source of the denervated areas, the overall number of large neurons is significantly smaller than in normal conditions. Thus, to compensate for the smaller number, the axonal branching might increase, which might underlie the abnormal activity of these neurons, as discussed above. Therefore, a decrease in the terminal arborization by increasing the number of reinnervating cells might decrease abnormal input from peripheral non-nociceptive neurons and, thus, affects the development of neuropathic pain. Another explanation could be that the disruption in the small-to-large cell ratio, which also coincides with the development of neuropathic pain, might lead to aberrations in the orchestrated input into CNS, leading to the abnormal activity of the pain-related spinal cord circuitry and central sensitization. Here, we show that treatment with ouabain increased the number of DRG neurons innervating the previously denervated skin without affecting an abnormal small-to-large cell ratio. This effect was sufficient to decrease neuropathic symptoms. These results imply that a reduced number of innervating cells, but not the small-to-large ratio of reinnervating neurons, is the key factor in the development of neuropathic pain. Continuing with this line of thought, an increase in the number of reinnervating neurons would, either by decreasing their axonal arborization or by other homeostatic plasticity mechanisms [30], reduce abnormal excitability of peripheral neurons, thus alleviating neuropathic pain.

Facilitating the innervation and examining whether it would reduce neuropathic pain would be one of the methods to casually connect between the decrease in innervating cells and the development of neuropathic pain. To that end, we used ouabain, which increased the number of reinnervating cells and, indeed, showed that it leads to a reduction of mechanical allodynia and hyperalgesia. There is no direct evidence of the effect of ouabain in DRG neurons. Moreover, a high dose of ouabain, which is sufficient for blocking Na, K-ATPase pump activity, was shown to inhibit neuronal sprouting [31]. However, recent studies have demonstrated that Na, K-ATPase can also function as a receptor and bind ouabain in low doses leading to changes in neuronal activity [32] and activating a variety of intracellular signaling cascades [33,34,35]. The application of low doses of ouabain was previously shown to improve recovery following brain injury [19]. Moreover, low doses of ouabain facilitated dendritic branching in hippocampal neurons [20]. These results, together with the data showing the ouabain-induced increase in the number of DRG neurons labeled with AF488 and AF647, which were injected into initially denervated areas (Figure 6, here), support the notion that the low doses of ouabain used here promoted sprouting of the neighboring neurons into denervated areas.

## 5. Conclusions

We have used a multi-spectral retrograde labeling-based approach to discover changes in the innervation patterns of skin that was initially denervated, during the development of neuropathic pain symptoms. This approach allowed for us to identify the DRG neurons which participate in reinnervation and to analyze how the number and type of innervating DRG neurons changes in correlation with developing neuropathic pain. The development of neuropathic pain was accompanied by an overall decrease in the number of DRG neurons and an increase in the fraction of large DRG neurons innervating initially denervated skin. Ouabain reversed the decrease in the number of innervating DRG neurons and alleviated neuropathic pain symptoms. These data suggest that the changes in innervation pattern following nerve injury, mainly the decrease in the number of innervating neurons, is a key factor in the development of nerve-injury induced pain. Therefore, the facilitation of reinnervation by therapies that are similar to ouabain, used here, may provide a new avenue for the development of neuropathic pain therapies. Importantly, our results showing that changes in skin innervation patterns affect neuropathic pain, emphasize, yet again, the role of the peripheral nervous system in the development and maintenance of neuropathic pain.
